# ModBase, a database of annotated comparative protein structure models and associated resources

**DOI:** 10.1093/nar/gkt1144

**Published:** 2013-11-23

**Authors:** Ursula Pieper, Benjamin M. Webb, Guang Qiang Dong, Dina Schneidman-Duhovny, Hao Fan, Seung Joong Kim, Natalia Khuri, Yannick G. Spill, Patrick Weinkam, Michal Hammel, John A. Tainer, Michael Nilges, Andrej Sali

**Affiliations:** ^1^Department of Bioengineering and Therapeutic Sciences, California Institute for Quantitative Biosciences, Byers Hall at Mission Bay, Office 503B, University of California at San Francisco, 1700 4th Street, San Francisco, CA 94158, USA, ^2^Department of Pharmaceutical Chemistry, California Institute for Quantitative Biosciences, Byers Hall at Mission Bay, Office 503B, University of California at San Francisco, 1700 4th Street, San Francisco, CA 94158, USA, ^3^Graduate Group in Biophysics, University of California at San Francisco, CA 94158, USA, ^4^Structural Bioinformatics Unit, Structural Biology and Chemistry department, Institut Pasteur, 25 rue du Docteur Roux, 75015 Paris, France, ^5^Université Paris Diderot-Paris 7, école doctorale iViv, Paris Rive Gauche, 5 rue Thomas Mann, 75013 Paris, France, ^6^Physical Biosciences Division, Lawrence Berkeley National Laboratory, Berkeley, CA 94720, USA, ^7^Department of Molecular Biology, Skaggs Institute of Chemical Biology, The Scripps Research Institute, La Jolla, CA 92037, USA, ^8^Life Sciences Division, Department of Molecular Biology, Lawrence Berkeley National Laboratory, Berkeley, CA 94720, USA

## Abstract

ModBase (http://salilab.org/modbase) is a database of annotated comparative protein structure models. The models are calculated by ModPipe, an automated modeling pipeline that relies primarily on Modeller for fold assignment, sequence-structure alignment, model building and model assessment (http://salilab.org/modeller/). ModBase currently contains almost 30 million reliable models for domains in 4.7 million unique protein sequences. ModBase allows users to compute or update comparative models on demand, through an interface to the ModWeb modeling server (http://salilab.org/modweb). ModBase models are also available through the Protein Model Portal (http://www.proteinmodelportal.org/). Recently developed associated resources include the AllosMod server for modeling ligand-induced protein dynamics (http://salilab.org/allosmod), the AllosMod-FoXS server for predicting a structural ensemble that fits an SAXS profile (http://salilab.org/allosmod-foxs), the FoXSDock server for protein–protein docking filtered by an SAXS profile (http://salilab.org/foxsdock), the SAXS Merge server for automatic merging of SAXS profiles (http://salilab.org/saxsmerge) and the Pose & Rank server for scoring protein–ligand complexes (http://salilab.org/poseandrank). In this update, we also highlight two applications of ModBase: a PSI:Biology initiative to maximize the structural coverage of the human alpha-helical transmembrane proteome and a determination of structural determinants of human immunodeficiency virus-1 protease specificity.

## INTRODUCTION

The genome sequencing efforts provide us with the complete genetic blueprints of thousands of organisms, including many eukaryotic genomes. We are now faced with the challenge of assigning, investigating and modifying the functions of proteins encoded by these genomes. This task is generally facilitated by the knowledge of the 3D protein structures, which are best determined by experimental methods such as X-ray crystallography and nuclear magnetic resonance-spectroscopy. While the number of experimentally determined structures deposited in the Protein Data Bank (PDB) (1) increased by nearly 40% to ∼93 000 in the past 3 years (September 2013), the number of sequences in the comprehensive sequence databases, such as UniProtKB (2) and GenPept (3), continues to grow even more rapidly; for example, the number of sequences in UniProtKB has now reached >41 million, compared with 12 million only 3 years ago. Therefore, protein structure prediction is essential to bridge this gap. The need for accurate models can frequently be met by homology or comparative modeling (4–13). Comparative modeling is carried out in four sequential steps: identifying known structures (templates) related to the sequence to be modeled (target), aligning the target sequence with the templates, building models and assessing the models. For this reason, comparative modeling is only applicable when the target sequence is detectably related to a known protein structure.

As more proteins are modeled, web-accessible resources that assist biologists in evaluating and analyzing models become increasingly useful. Here, we describe the current state of the ModBase database of comparative protein structure models, the ModWeb comparative modeling web-server and several new associated resources, including web-servers that use SAXS data in the context of comparative modeling: The AllosMod server for modeling ligand-induced protein dynamics (http://salilab.org/allosmod) (14), the AllosMod-FoXS server for predicting the ensemble of conformations that best fit a given SAXS profile (http://salilab.org/allosmod-foxs) (Weinkam *et al.* in preparation), the FoXSDock server that performs protein–protein docking filtered by a SAXS profile (http://salilab.org/foxsdock) (15), the SAXS Merge server for merging SAXS profiles (http://salilab.org/saxsmerge) (Spill *et al.* accepted) and the Pose & Rank server for scoring protein–ligand complexes based on a statistical potential (http://salilab.org/poseandrank) (16). Finally, we highlight applications of ModBase models to maximize the structural coverage of the human α-helical transmembrane proteome in a PSI:Biology effort; and to an analysis of structural determinants of human immunodeficiency virus-1 (HIV-1) protease specificity.

## CONTENTS

### Model generation by comparative modeling (Modeller and ModPipe)

Models in ModBase are calculated using our automated software pipeline for comparative protein structure modeling, ModPipe (17). ModPipe relies mostly on modules of Modeller (18) as well as fold assignment and sequence-structure alignment by PSI-BLAST (19) and the HHSuite modules HHBlits (20) and HHSearch (21). To be able to process a large number of sequences, it is implemented on a Linux cluster.

ModPipe uses sequence–sequence (22), sequence–profile (19,23) and profile–profile (5,24) methods for fold assignment and target–template alignment, using a promiscuous E-value threshold of 1.0 to increase the likelihood of identifying the best available template structure. In addition to the previously implemented profile methods (Modeller’s Build-Profile and PPScan, and PSI-BLAST), we recently added an option to use HHBlits and HHSearch. These will be included in the next public release of ModPipe (2.3.0, expected December 2013). Alignments created by any of these methods can cover the complete target sequence, or only a segment of it, depending on the availability of suitable PDB templates. With the added functionality of HHBlits and HHSearch, some ModPipe models are now based on multiple templates.

To increase efficiency, the available target–template alignments are filtered by sequence identity (ModPipe template option: TOP): if the highest target–template sequence identity is ≤40%, ModPipe selects alignments for all detected templates. Otherwise, the selection only contains alignments for each target–template alignment that is created in a 20% sequence identity window starting from the highest sequence identity. For each selected target–template alignment, 10 models are calculated (18), and the model with the best value of the DOPE statistical potential (25) is selected and then evaluated by several additional quality criteria: (i) target–template sequence identity, (ii) GA341 score (26), (iii) Z-DOPE score (25), (iv) MPQS score (ModPipe quality score) (27) and (v) TSVMod score (28). The models that score best with at least one of these quality criteria are selected for further filtering. If >30 residues of a target sequence are not covered by a selected model, additional models are selected even if they do not score best with at least one of the quality criteria. Finally, only the models with quality criteria values above specified thresholds or with an E-value <10^−^^4^ are included in the final model set.

A key feature of the pipeline is that the validity of sequence–structure relationships is not prejudged at the fold-assignment stage; instead, sequence–structure matches are assessed after the construction of the models and their evaluation. This approach enables a thorough exploration of fold assignments, sequence–structure alignments and conformations, with the aim of finding the model with the best evaluation score, at the expense of increasing the computational time significantly; for some sequences, a few thousand models can be calculated. For sequences with high-quality templates, the optional ‘TOP’ keyword can reduce the amount of computer time by up to 60%.

The source code for ModPipe is freely accessible under the Gnu Public license (http://salilab.org/modpipe). The binary code for Modeller is also available freely to academics for a number of different operating systems (http://salilab.org/modeller).

### Statistically optimized atomic potentials (SOAP) for assessing protein interfaces and loops

Both loop modeling and protein–protein docking require accurate scoring functions for selecting the most accurate sampled models. Statistically Optimized Atomic Potentials (SOAP)-PP and SOAP-Loop are atomic statistical potentials for assessing protein interfaces and loops, respectively (http://salilab.org/SOAP, also available in Modeller) (29). They were derived using a Bayesian framework for inferring SOAP. When using SOAP-PP for scoring protein–protein docking models, a near-native model is within the top 10 scoring models in 52% of the PatchDock decoys (30), compared with 23 and 27% for the state-of-the-art ZRANK (31) and FireDock (32) scoring functions, respectively. Similarly, for modeling 12-residue loops in the PLOP benchmark (33), the average main-chain root-mean-square-deviation (RMSD) of the best-scored conformations by SOAP-Loop is 1.5 Å, close to the average RMSD of the best-sampled conformations (1.2 Å) and significantly better than that selected by the Rosetta (34) (2.1 Å), DFIRE (35) (2.3 Å), DOPE (2.5 Å) (25) and PLOP scoring functions (3.0 Å). The SOAP-PP score is used by our AllosMod-FoXS server (below). We are incorporating SOAP scores into the modeling and model assessment modules of ModPipe.

### ModBase model sets

Models in ModBase are organized in datasets. Because of the rapid growth of the public sequence databases, we concentrate our efforts on adding datasets that are useful for specific projects, rather than attempt to model all known protein sequences based on all detectably related known structures. Currently, ModBase includes a model dataset for each of 65 complete genomes, as well as datasets for all sequences in the Structure Function Linkage Database (SFLD) (36), and for the complete SwissProt/TrEMBL database as of 2005 (http://salilab.org/modbase/statistics). Additionally, available models for new SFLD sequences are added weekly. Together with other project-oriented datasets, ModBase currently contains ∼29 million reliable models for domains in 4.7 million unique sequences. The ‘Nominate a modelome!’ feature allows community users to request modeling of additional complete genomes as our computational resources allow. This feature has been used, for example, to support the Tropical Disease Initiative (http://tropicaldisease.org) (37–40)

### ModWeb: comparative modeling web-server

The ModWeb comparative modeling web-server is an integral module of ModBase (http://salilab.org/modweb) (17). In the default mode, ModWeb accepts one or more sequences in the FASTA format, followed by calculating and evaluating their models using ModPipe based on the best available templates from the PDB. Alternatively, ModWeb also accepts a protein structure as input (template-based calculation), calculates a multiple sequence profile and identifies all homologous sequences in the UniProtKB database, followed by modeling these homologs based on the user-provided structure. This alternative protocol is a useful tool for measuring the impact of new structures, such as those generated by structural genomics efforts (41). Moreover, new members of sequence superfamilies with at least one known structure can be identified (42).

In addition to anonymous access, registered users get unified access to all their ModWeb datasets and can submit template-based calculations.

## ASSOCIATED RESOURCES

A number of web services are associated with ModBase. Some of these are tightly integrated with ModBase, whereas others contain data that are derived through ModBase [e.g. single-nucleotide polymorphism (SNP) annotations created by LS-SNP (43)]. We already described the interactions of ModBase with the ModLoop server for loop modeling in protein structures (http://salilab.org/modloop) (44), the PIBASE database of protein–protein interaction (http://salilab.org/pibase) (45), the DBAli database of structural alignments (http://salilab.org/dbali) (46,47), the LS-SNP database of structural annotations of human non-synonymous SNPs (http://salilab.org/LS-SNP) (43,48,49), the SALIGN server for multiple sequence and structure alignment (http://salilab.org/salign) (27,50), the ModEval server for predicting the accuracy of protein structure models (http://salilab.org/modeval) (27), the PCSS server for predicting which peptides bind to a given protein (http://salilab.org/pcss) (27) and the FoXS server for calculating and fitting small angle X-ray scattering profiles (http://salilab.org/foxs) (27,51). Here, we describe several new servers that interact with ModBase.

### AllosMod: a web-server for modeling ligand-induced protein dynamics

Conformational transitions of biomolecules are key to many aspects of biology. These dynamic changes span a broad range of time and size scales, and include protein folding, aggregation, induced fit and allostery.

The **AllosMod** web server (http://salilab.org/allosmod) predicts conformational changes that occur in the native ensemble, such as allosteric conformational transitions. The input is one or more macromolecular coordinate files (including DNA, RNA and sugar molecules) and the corresponding sequence(s). The output is a set of molecular dynamics trajectories based on a simplified energy landscape. The documentation includes analysis examples to help the user in interpreting the expected output. Carefully designed energy landscapes allow efficient molecular dynamics sampling at constant temperatures, thereby providing ergodic sampling of conformational space. AllosMod energy landscapes are constructed using contacts in crystal structure(s) to define the energetic minima. This model is referred to as a structure-based or Gō model (52–54). The energy landscapes are sampled using many short constant temperature molecular dynamics simulations. Sampling occurs quickly, even for large systems with up to 10 000 residues, because the simplified landscapes can be stored in memory. The user can also download Python scripts necessary to run and modify the simulations, which are performed using Modeller (18).

The capabilities of the AllosMod server have been demonstrated in a study of allosteric systems with known effector bound and unbound crystal structures (14,55). Effector bound and unbound simulations are performed using a landscape with a single minimum for the interactions in the effector binding site, corresponding to the bound or unbound structure and dual minima for interactions in the rest of the protein, corresponding to the bound and unbound structures. AllosMod can also be used to predict coupling (i.e. ΔΔG) between a mutation site and the effector binding site.

### A family of web-servers for computation and application of SAXS profiles

SAXS is a common technique for low-resolution structural characterization of molecules in solution (56,57). SAXS experiments determine the scattering intensity of a molecule as a function of spatial frequency, resulting in a SAXS profile that can be easily converted into the approximate distribution of atomic distances in the measured system. The experiments can be performed with the protein sample in solution, and usually take only a few minutes on a well-equipped synchrotron beamline (57). Here, we describe new features of the FoXS server for calculating and fitting SAXS profiles, the AllosMod-FoXS server that predicts the structural ensemble that best fits a given SAXS profile, the FoXSDock server that performs protein–protein docking filtered by a SAXS profile and the SAXS Merge server for merging SAXS profiles measured at different concentrations and exposure times.

**Figure 1.**
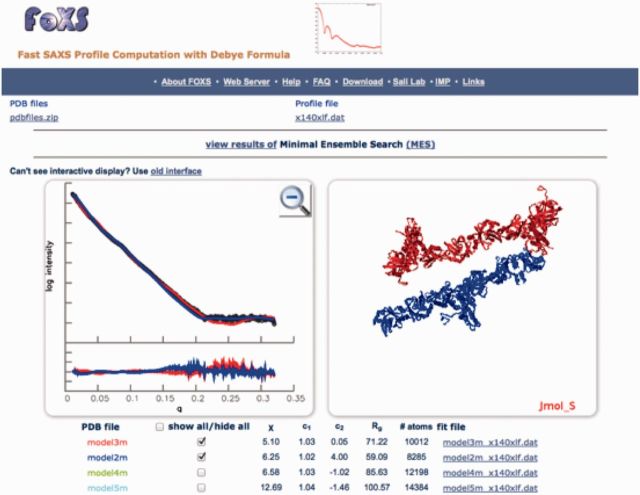
The computed profiles for filament models of the XLF–XRCC4 complex (63) are fitted to the experimental SAXS profile with FoXS. The interactive user interface displays the profiles in the left and the models in the right using the same color for each model/profile pair. The table below the panels displays the fit parameters and includes buttons to simultaneously show or hide each model/profile pair. Clicking on Minimal Ensemble Search (MES) results (above the display panel) takes the user to the MES output page.

FoXS (http://salilab.org/foxs) is a rapid and accurate server for calculating a SAXS profile of a given molecular structure (51). The input is one or more macromolecular coordinate files or PDB codes and an experimental profile. The output is a calculated SAXS profile for each input structure, fitted onto the experimental profile. The method explicitly computes all inter-atomic distances and models the first solvation layer based on solvent accessibility. FoXS was tested on 11 protein, 1 DNA and 2 RNA structures, revealing superior accuracy and speed versus CRYSOL (58), AquaSAXS (59), the Zernike polynomials-based method (60) and Fast-SAXS-pro (61). In addition, we demonstrated a significant correlation of the SAXS score with the accuracy of a structural model (62). We have recently updated the server to an interactive user interface; profiles are displayed via an HTML5 canvas element and structures are shown in a Jmol window (Figure 1). If the user uploads multiple structures, the server automatically performs the minimal ensemble computation with Minimal Ensemble Search (MES) (64).

AllosMod-FoXS (http://salilab.org/allosmod-foxs) is a server that predicts the structural ensemble that best fits a given SAXS profile. The input is one or more macromolecular coordinate files, the corresponding sequence(s) and an ‘experimental’ SAXS profile. The output is the structural ensemble that best fits the input SAXS profile. The server relies on AllosMod conformational sampling (14), FoXS calculations of theoretical SAXS profiles, minimal ensemble computation with MES (64) and the SOAP-PP score. The server was motivated to describe conformational changes in proteins, such as the allostery, based on both modeling considerations (as represented by AllosMod) and experimental SAXS data (as represented by FoXS).

The AllosMod-FoXS server uses various sampling algorithms in AllosMod to generate structures that are directly entered into FoXS. Because FoXS explicitly computes all inter-atomic distances and models the first solvation layer based on solvent accessibility, it can be used to score the similarity of the experimental SAXS profile to the predicted SAXS profiles corresponding to structures from the AllosMod simulations. In addition to the FoXS score, each conformation is assessed for structural quality, using the SOAP-PP score. These two scores are combined to predict structures that collectively best explain the experimental SAXS profile.

FoXSDock (http://salilab.org/foxsdock) is a web server that uses SAXS profiles to filter the models produced by protein–protein docking. It accepts as input structures of two docked proteins and an experimental SAXS profile of their complex. The output is a set of docking models and their calculated SAXS profiles fitted onto the experimental profile. Although many structures of single protein components are becoming available, structural characterization of their complexes remains challenging. Although general, protein–protein docking methods suffer from large errors because of protein flexibility and inaccurate scoring functions. However, when additional information, such as a SAXS profile, is available, it is possible to significantly increase the accuracy of the computational docking.

FoXSDock combines rigid global docking by PatchDock, filtering of the models based on the SAXS profile and interface refinement by FireDock (15). The approach was benchmarked on 176 protein complexes with simulated SAXS profiles, as well as on 7 complexes with experimentally determined SAXS profiles (30). When induced fit is <1.5 Å interface C_α_ RMSD and the fraction of residues missing from the component structures is <3%, FoXSDock can find a model close to the native structure within the top 10 predictions in 77% of the cases; in comparison, docking alone succeeds in only 34% of the cases.

SAXS Merge (http://salilab.org/saxsmerge) is a web server that uses automated statistical methods to merge SAXS profiles determined at different concentrations and exposure times. High-throughput SAXS data collection requires robust, accurate and automated tools for data processing and merging (57,65). However, SAXS data are generally processed highly subjectively, often manually with the aid of the PRIMUS software package (66). The operation requires an experienced user who can manually inspect each profile to be merged and decide whether the SAXS profiles agree or not. The SAXS Merge web-server alleviates user intervention through an automated and statistically principled merging procedure based on a Bayesian approach (Spill *et al.* submitted). The SAXS Merge web server was successfully validated on a benchmark of 16 SAXS datasets. The input file consists only of the buffer-subtracted SAXS profiles in a common three-column text format. The output comprises (i) a list of individual q points with associated source profiles, (ii) an estimate of the mean profile, along with a 95% Bayesian credible interval and (iii) the most suitable parametric mean function for the resulting profile, an estimate of the noise level in the pooled dataset. The output is visualized interactively through the web-browser and can also be downloaded.

### Pose & rank: a web-server for scoring protein–ligand complexes

Molecular recognition between proteins and ligands plays an important role in many biological processes. Predicting the structures of protein–ligand complexes and finding ligands by virtual screening of small molecule databases are two long-standing goals in molecular biophysics and medicinal chemistry. Solving both problems requires the development of an accurate and efficient scoring function to assess protein–ligand interactions.

The Pose & Rank web server (http://salilab.org/poseandrank) (16) provides access to two atomic distance-dependent statistical scoring functions based on probability theory that can be used in protein–ligand docking: The PoseScore was optimized for recognizing native binding geometries of ligands from other poses, and the RankScore was optimized for distinguishing ligands from non-binding molecules. The server accepts as input a coordinate file of the target protein structure in the PDB format and docking poses of small molecules. The output is a list of scores for each protein–small molecule complex. PoseScore ranks a near-native binding pose the best, top 5 and top 10 for 88%, 97% and 99% of targets, respectively. RankScore improves the overall ligand enrichment (logAUC) and early enrichment (EF1) scores computed by DOCK 3.6 (67) for 68% and 74% of targets, respectively. The Pose & Rank resource can contribute to many applications, such as selecting ligand candidates from virtual screening for experimental testing, predicting the binding geometries for known ligands and suggesting binding site mutations that alter the ligand binding properties and consequently protein functions.

## APPLICATION EXAMPLES

### Coordinating the impact of structural genomics on the human α-helical transmembrane proteome

With the recent successes in determining membrane protein structures, we explored the tractability of determining representatives for the entire human transmembrane proteome (68) (http://salilab.org/membrane). This proteome contains 2925 unique integral α-helical transmembrane domain sequences that cluster into 1201 families sharing >25% sequence identity. We assessed the modeling coverage by processing all sequences through ModPipe, and analyzing the resulting ModBase dataset. We then clustered all sequences [BlastClust(69)], annotated them with cluster size, modeling coverage and number of predicted transmembrane helices. Finally, we explored several target selection strategies. Structures of 100 optimally selected targets would increase the fraction of modelable human alpha-helical transmembrane domains from 26 to 58%, thus providing structure/function information not otherwise available.

To leverage the results of this study, the PSI:Biology Network (http://www.nigms.nih.gov/Research/FeaturedPrograms/PSI/psi_biology/), including high-throughput and membrane PSI centers as well as the Structural Genomics Consortium, is attempting to express nearly 100 human transmembrane proteins using their standard high-throughput methods. The goal of this survey is to determine which methods best express certain classes of transmembrane proteins. The sequences of our previous analysis were further annotated by fraction of predicted disordered regions (70,71), number of glycosylation sites (2,72,73), clone availability (74–76), HUGO annotations (77), sequence length and several additional metrics. Eighty-six targets were hand-picked from the largest clusters to represent a diverse selection of human membrane proteins with maximum coverage of the transmembrane proteome. Cloning, expression and solubility experiments of these targets using the pipelines of the 10 participating research groups are currently in progress. Participants also use shared and individual sets of six controls. A standard method will be used by all to visualize the protein bands to quantify yield. A final full comparison will determine the most successful methods for each representative transmembrane protein. Progress of the survey is cataloged by the portal of the Protein Structure Initiative Structural Biology Knowledgebase [PSI SBKB (78); http://hmpps.sbkb.org/] and will be accessible to the public after the conclusion of the experiment. A final publication will summarize the survey’s findings.

### Structural determinants of HIV-1 protease

The maturation of the HIV virion is facilitated by the cleavage of the Gag and Pol polyproteins (79). A homodimeric aspartic protease (HIV-1 protease) catalyzes these processing events at 10 non-homologous sites and is the target of some of the most effective antiretroviral drugs (80–82). These sites are eight amino acid residues in length; the cleavage occurs between the third and fourth residues (83–86). In addition to processing viral proteins, HIV-1 protease cleaves several human proteins during infection, such as the eukaryotic translation initiation factor 3 subunit D (eIF3D) (87–90).

**Figure 2.**
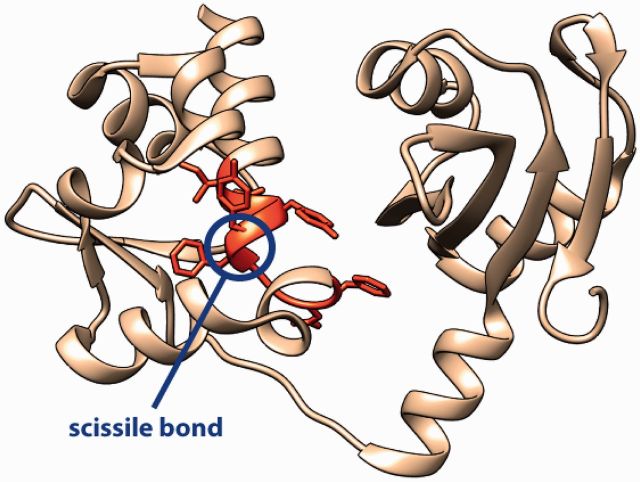
Cleavage of human proteins by the HIV-1 protease: crystal structure of the N-terminal domain of human Lupus La protein (92) (left). Residues of the cleavage site (Ile-Asp-Tyr-Tyr-Phe-Gly-Glu-Phe) are shown in orange. Scissile bond between Tyr and Phe in the alpha-helix is cleaved by the HIV-1 protease *in vitro*.

To predict cleavage sites in human proteins, we began by examining sequence and structural features of >120 cellular substrates of HIV-1 protease that were recently identified *in vitro* (91) (for an example, see Figure 2). First, every residue of the cleaved and non-cleaved octapeptides was encoded using >512 physicochemical amino acid indices (93,94). To account for cooperativity between residues in different positions of the octapeptide, frequencies of dipeptides and gapped dipeptides (i.e. two specific residues separated by any residue) were also used to train machine learning algorithms for binary classification. Second, a greedy feature selection procedure was applied to determine features of octapeptides important for protease activity. Interestingly, although features encoding known viral cleavage motif ELLE were important for classification, most discriminating features encode structural preferences of amino acid residues in the second and fifth positions of the octapeptide. Therefore, we created a ModBase dataset of 405 models for 118 human proteins cleaved *in vitro*. PSI-Pred (95) was used to predict secondary structure elements for protein regions without templates. Analysis of the structural models showed the enrichment of alpha+beta protein class (SCOP ID = 53 931) among cleaved proteins and coiled secondary structure (∼41%) among cleaved sites. We added structure-based descriptors of cleaved and non-cleaved sites to the sequence-based features and assessed classifiers’ performance in a 5-fold cross-validation procedure. The average area under the receiver operating characteristic curve for the classifier trained with the Random Forest algorithm(96) was 0.965 (72% sensitivity and 98% specificity) and the entire human proteome was scanned for putative human substrates of the HIV-1 protease. We are currently experimentally validating several of the predicted cleavage sites.

## ACCESS AND INTERFACE

### Direct access

The main access to ModBase is through its web interface at http://salilab.org/modbase, by querying with UniprotKB (2,3) and GI (97) identifiers, gene names, annotation keywords, PDB(1) codes, dataset names, organism names, sequence similarity to the modeled sequences [BLAST(19)] and model-specific criteria such as model reliability, model size and target–template sequence identity. Additionally, it is possible to retrieve coordinate files and alignment files of all models for a specific sequence as text files. Metadata for all current ModBase models (updated weekly), all genome datasets and several additional project specific datasets, are also available from our FTP server (ftp://salilab.org/databases/modbase/projects).

**Figure 3.**
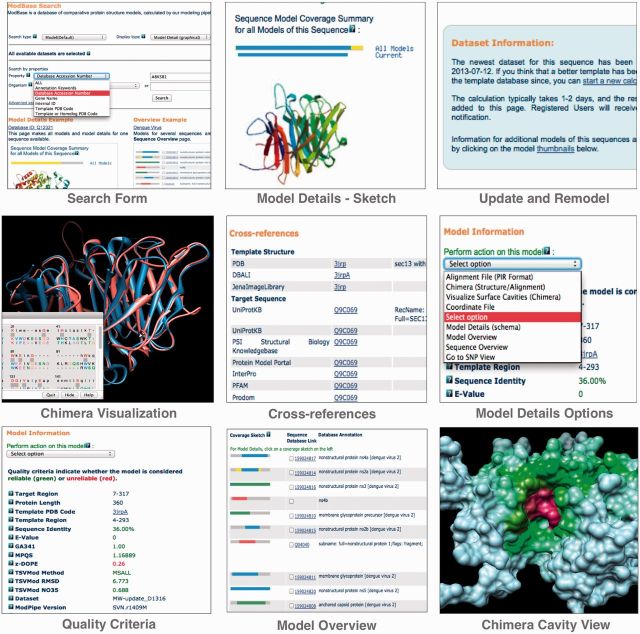
ModBase interface elements. **Search Form**: search options are available through the pull-down menu. A quick overview of the available representations is displayed below the search form. **Model Details Sketch**: the Model details page provides information for all models of a given sequences. The sketch comprises two parts: the model coverage sketch that indicates the sequence coverage by all models (top line) and the sequence coverage by the current model (second line), and a ribbon diagram of the current model. Other models are available via thumbprints. **Update and Remodel**: this box shows the date of the last modeling calculation for the current sequence, and allows the user to request an update. **Chimera Visualization**: the visualization includes the model and template structures and the alignment. **Cross-references**: links to the PMP, UniProtKB, Genbank, UCSC Genome Browser and other databases. **Model Details Options**: the pull-down menu switches between representations and allows downloads of coordinate and alignment files. **Quality Criteria**: red indicates unreliable, green reliable. **Model Overview**: a different representation for several sequences gives a quick overview on modeling coverage and quality. **Chimera Cavity View**: visualizes cavities predicted by ConCavity.

The output of a search is displayed on pages with varying amounts of information about the modeled sequences, template structures, alignments and functional annotations. Output examples from a search resulting in one model are shown in Figure 3. A ribbon diagram of the model with the highest target–template sequence identity is displayed by default, together with some details of the modeling calculation. Ribbon thumbprints of additional models for this sequence link to corresponding pages with more information. Ribbon diagrams are generated on the fly using Molscript (98) and Raster3D (99). A pull-down menu provides links to additional functionalities: the SNP module; retrieval of coordinate and alignment files; molecular visualization by UCSF Chimera (100) that allows the user to display template and model coordinates together with their alignment; and Chimera visualization of predicted cavities [ConCavity (101)]. If mutation information is available for a protein sequence, links to the details are provided in the cross-references section. Additionally, cross-references to various other databases, including PDB (102), UniProtKB (103), the UCSC Genome Browser (104), EBI’s InterPro (105), PharmGKB (106) and SFLD (36) are given. Other ModBase pages provide overviews of more than one sequence or structure. All ModBase pages are interconnected to facilitate easy navigation between different views.

### Access through external databases

#### The Protein Model Portal

The Protein Model Portal (PMP) has become a valuable option for accessing ModBase models (http://proteinmodelportal.org) (107). The PMP is a single point of entry for accessing protein structure models from a number of different databases. PMP queries all participating source model databases and serves the user with the model coordinates, alignments and quality criteria from a central location. It has been developed as a module of the Protein Structure Initiative Knowledgebase (PSI KB) (79,108). The PMP provides a flexible search interface for all deposited models, quality estimation, cross-links to other sequence and structure databases, annotations of sequences and their models, a central point of entry to comparative modeling servers (including ModWeb) and quality estimation servers (including ModEval) and detailed tutorials on all aspects of comparative modeling. Currently, the PMP retrieves ∼450 000 ModBase model coordinate files each week from ModBase.

A sister web-service to PMP, CAMEO (http://cameo3d.org) (107) continuously evaluates the accuracy and reliability of several comparative protein structure prediction servers in a fully automated manner. The ModWeb server currently participates in the testing mode, and is expected to move into the production mode in the first quarter of 2014.

#### Access through external databases

ModBase models in academic and public datasets are also directly accessible from several databases, including the PMP (107), UniProtKB (109), PIR’s iProClass (103), EBI’s InterPro (105), the UCSC Genome Browser (104), PubMed (LinkOut) (110), PharmGKB (106) and SFLD (36).

## FUTURE DIRECTIONS

ModBase will grow by adding models calculated on demand by external users (using ModWeb) as well as our own calculations of model datasets that are needed for our research projects (using ModPipe, ModWeb or Modeller). These updates will reflect improvements in the methods and software used for calculating the models as well as new template structures in the PDB and new sequences in UniProtKB. In the future, we expect that most of the users will access ModBase models through the PMP.

## CITATION

Users of ModBase are requested to cite this article in their publications.

## FUNDING

National Institutes of Health [U54 GM094662, U54 GM094625, U54 GM093342, MINOS R01GM105404 to J.A.T. and M.H.]; Sandler Family Supporting Foundation (A.S.); Department of Energy Lawrence Berkeley National Lab IDAT program (to J.A.T. and M.H.); European Union [FP7-IDEAS-ERC 294809 to M.N.]. The authors thank Tom Ferrin and the UCSF Resource for Biocomputing, Visualization and Informatics for making UCSF Chimera (supported by [NIGMS P41-GM103311]) available to the ModBase database and tools. Funding for open access charge: NIH.

*Conflict of interest statement*. None declared.
